# *KRAS* and *TP53* Circulating Tumor DNA are Prognostic in Localized Pancreatic Cancer

**DOI:** 10.1245/s10434-026-20447-0

**Published:** 2026-08-26

**Authors:** Krishay Sridalla, Dominic J. Vitello, Madison Cox, Larissa Masnyk, Amy Wells, Alex Horowitz, Dhavan Shah, Jasmine K. Machhi, Vishvetha Rengaraju, Anastasia Chibucos, Claire Shen, Lauren M. Janczewski, John Abad, Kevin Dawravoo, David J. Bentrem, Lawrence J. Jennings, Qiang Zhang, Akhil Chawla

**Affiliations:** 1 Division of Surgical Oncology, Department of Surgery, Northwestern University Feinberg School of Medicine, Chicago, IL USA; 2 Northwestern Quality Improvement, Research & Education in Surgery,, Department of Surgery, Northwestern University Feinberg School of Medicine, Chicago, IL USA; 3 Northwestern Medicine Cancer Centers, Northwestern Medicine Regional Medical Group, Winfield, IL USA; 4 Division of Medical Oncology, Department of Medicine, Northwestern University Feinberg School of Medicine, Chicago, IL USA; 5 Robert H. Lurie Comprehensive Cancer Center of Northwestern University, Chicago, IL USA; 6 Division of Genomic Pathology, Department of Pathology, Northwestern University Feinberg School of Medicine, Chicago, IL USA; 7 Circulating Tumor Cell Core Facility, Robert H. Lurie Comprehensive Cancer Center, Northwestern University Feinberg School of Medicine, Chicago, IL USA

**Keywords:** Circulating tumor DNA, Pancreatic ductal adenocarcinoma, Next-generation sequencing, Biomarker, Neoadjuvant chemotherapy

## Abstract

**Background:**

Pancreatic ductal adenocarcinoma (PDAC) carries high mortality despite neoadjuvant chemotherapy (NAC) and surgical resection, creating an urgent need for prognostic biomarkers to guide treatment. We aimed to determine whether circulating tumor DNA (ctDNA) detected by next-generation sequencing (NGS) predicts survival in patients with localized PDAC.

**Methods:**

Patients with localized PDAC were prospectively enrolled at an academic institution between October 2020 and November 2024 (NCT04616131). Patients underwent next-generation sequencing (NGS), with blood samples collected at diagnosis, post-NAC, and post-resection. Total, *KRAS*-specific, and *TP53*-specific ctDNA detection rates were quantified. Associations with overall survival (OS) were evaluated by using Kaplan–Meier curves and multivariable Cox proportional hazards models adjusting for key clinical covariates.

**Results:**

We collected 219 samples from 113 patients across three treatment timepoints. Mutant *KRAS* ctDNA was detected in 19% at diagnosis, decreased to 4.6% after NAC (19% vs. 4.6%, *p* = 0.010), and was detected in 8.2% after resection. Total ctDNA detection at diagnosis predicted lower odds of proceeding to resection (adjusted odds ratio [aOR] 0.38, 95% confidence interval [CI] 0.14–0.97, *p* = 0.049). *KRAS* and *TP53* co-occurrence at diagnosis independently predicted shorter OS (adjusted hazard ratio 5.66, 95% CI 2.43–13.18, *p* < 0.001). Increases in total ctDNA maximum variant allele frequency over treatment also predicted shorter OS (adjusted hazard ratio 1.14, 95% CI 1.01–1.28, *p* = 0.028).

**Conclusions:**

In a large prospective cohort, tumor-agnostic NGS-based ctDNA detection demonstrated prognostic value in patients with localized PDAC undergoing NAC and surgery. These findings suggest ctDNA can inform treatment monitoring and risk stratification, supporting further evaluation in larger, multi-center studies to guide clinical decision-making.

**Supplementary Information:**

The online version contains supplementary material available at https://doi.org/10.1245/s10434-026-20447-0.

Pancreatic ductal adenocarcinoma (PDAC) is the third-leading cause of cancer-related death in the United States, with 80–85% of patients presenting with unresectable disease.^1,2^ Even among those who undergo surgery, outcomes remain poor, with recurrence rates approaching 77% and median recurrence-free survival of 11.7 months.^3^ Neoadjuvant chemotherapy (NAC) is increasingly used in localized PDAC to downstage tumors, improve R0 resection rates, and treat micrometastatic disease.^4–6^ Treatment response is typically assessed using carbohydrate antigen 19-9 (CA 19-9) and imaging, although both have limitations: CA 19-9 is affected by benign conditions and interpatient variability, while imaging often cannot distinguish viable tumor from post-treatment fibrosis.^7–10^

Circulating tumor DNA (ctDNA) is a noninvasive biomarker for disease monitoring, prognosis, and molecular profiling across cancers.^11–14^ In PDAC, ctDNA detection and clearance are prognostic, although most studies have focused on metastatic disease or used platforms with limited genomic coverage.^15–20^ Few prospective studies have evaluated ctDNA dynamics during NAC in localized PDAC using next-generation sequencing (NGS), which offers broader genomic profiling.^21,22^

We previously showed that ctDNA, particularly *KRAS* and *TP53*-specific ctDNA, can be detected throughout NAC using a tumor-uninformed NGS approach, with its presence at diagnosis predicting shorter progression-free survival (PFS).^23^ Building on these findings, we investigated ctDNA detectability and prognostic utility using a clinically validated, tumor-agnostic NGS platform in patients with localized PDAC undergoing NAC and surgery. We assessed total, *KRAS*, and *TP53*-specific ctDNA detection, maximum variant allele frequency (mVAF), and the prognostic and predictive value of ctDNA dynamics during treatment.

## Methods

### Study Design

This prospective observational cohort study was approved by the Northwestern University Institutional Review Board (NMHC IRB #21-012) and registered at ClinicalTrials.gov (NCT04616131). The study followed the Strengthening the Reporting of Observational Studies in Epidemiology (STROBE) guidelines, and all participants provided written informed consent.^24^

Enrollment occurred across the Northwestern Medicine health system between October 2020 and November 2024. Eligible patients were ≥18 years with biopsy- or cytology-confirmed PDAC, no radiographic evidence of metastases, and planned NAC (Supplemental Fig. 1). Patients with NGS testing at ≥1 timepoint were included in analysis.

At Northwestern, NAC is routinely recommended for medically fit patients with potentially operable PDAC, while those without venous involvement are individually considered for NAC or clinical trial enrollment. Institutional practice is typically 3 months of NAC followed by restaging and consideration of surgery, with radiation therapy for select patients with arterial involvement. All patients undergo multidisciplinary discussion prior to local therapy, with select patients continuing NAC for up to 6 months based on imaging and patient factors. Some patients do not complete the planned NAC regimen due to disease progression, declining performance status, or other clinical considerations. All cases were reviewed at multidisciplinary tumor boards, and management followed institutional standards independent of study participation.

### Sample Collection and Processing

Peripheral blood (≤50 mL) was collected at prespecified timepoints: diagnosis, post-NAC, and post-resection. Patients who did not complete NAC were included, with ctDNA assessed at available timepoints; missing posttreatment samples were considered nonclearance events. Analyses included all available samples per patient, as sample availability varied (Supplemental Fig. 2). Patients with hyperbilirubinemia underwent biliary decompression, and CA19-9 was measured once bilirubin was <3 mg/dL. Blood was collected in Streck Cell-Free DNA tubes and shipped for NGS analysis.

### Next-Generation Sequencing

Circulating tumor DNA was analyzed using NGS from blood collected in Streck tubes using commercial, tumor-agnostic assays. Patients 1–60 underwent testing with the 105-gene Tempus x|F panel (Tempus Labs; Supplemental Table 1).^25^ Patients 61–113 were analyzed with the 152-gene PredicineCARE™ assay (Predicine; Supplemental Table 2).^26^ The switch to PredicineCARE™ reflected adoption of a newer assay with broader genomic coverage, validated in ctDNA studies, and increasingly used clinically.^27,28^ Overall survival by assay is presented in Supplemental Fig. 3.

In this study, *KRAS* and *TP53*-specific ctDNA refer to ctDNA fragments harboring mutations in *KRAS* or *TP53*, respectively, while total ctDNA includes any detectable somatic alteration identified by the gene panel, including but not limited to *KRAS* and *TP53*. For a subset of patients, matched tumor tissue was obtained at diagnosis (fine-needle aspiration) or resection to compare with plasma NGS *KRAS* mutational profiles.

### Statistical Analysis

Analyses were conducted using R (v4.4.1). Demographic and clinical characteristics were compared by total ctDNA detection at diagnosis by using Fisher’s exact or χ^2^ tests for categorical variables and the Mann-Whitney *U* test for continuous variables. Multivariable logistic regression evaluated associations between baseline total ctDNA detection (binary) and receipt of resection (binary), adjusting for baseline resectability (resectable, borderline-resectable, locally advanced) and receipt of NAC (binary). Results were reported as adjusted odds ratios (aORs) with 95% confidence intervals (CI).

Follow-up duration was calculated from diagnosis to last contact or death. Kaplan-Meier curves were compared using the log-rank test. Multivariable Cox proportional hazards models included clinically relevant variables available at diagnosis. The model evaluating baseline total ctDNA detection adjusted for age, sex, clinical stage, pre-treatment CA 19-9, NAC receipt, and resection. Harrell’s C‑index was calculated from the Cox model and assessed the model’s discriminatory ability to rank patients by survival risk.

In this study, mVAF reflected the highest mutant allele frequency in ctDNA and serves as a proxy for tumor mutational burden.^29^ Total ctDNA mVAF reflected the maximum allele frequency of any variant detected, whereas *KRAS* ctDNA mVAF represented the maximum allele frequency of *KRAS* variants. For patients with detectable ctDNA at both timepoints, mVAF change was defined as post-NAC minus baseline mVAF. The cutoff for total ctDNA mVAF change was determined by log-rank testing with Bonferroni correction. A separate Cox model assessed change in total ctDNA mVAF from diagnosis to post-NAC as a continuous variable, adjusting for the same covariates except NAC and resection, as all patients included received NAC and limited sample size precluded adjustment for resection. Missing data were addressed with complete case analysis, and all tests were two-sided.^30^

## Results

### Patient and Treatment Characteristics

The cohort included 113 patients (Table 1). The majority were aged 60–79 years (71.7%), male (53.1%), and Caucasian (86.7%). Tumors were predominantly in the pancreatic head/uncinate process (71.7%). At diagnosis, clinical stage was most commonly IB (46.9%), followed by III (22.1%), IA (15.9%), and II (15.0%). By National Comprehensive Cancer Network (NCCN) criteria, 61.1% were resectable, 20.3% borderline-resectable, and 18.6% locally advanced, with corresponding resection rates of 82.1% (32/39), 64.9% (37/57), and 5.9% (1/17). Total ctDNA was detected in 53.3% of patients undergoing testing at diagnosis (56/105) and was not associated with baseline demographic or clinical characteristics.

**Table 1:** Baseline clinical and demographic characteristics of the study cohort by ctDNA detection status at diagnosis

Characteristic	Total	Total ctDNA Detected	Total ctDNA Not Detected	*p*
	n (%)^a^	n (%)	n (%)	
Total	113 (100)^b^	56 (49.56)	49 (43.36)	
*Age group*				
19–39	1 (0.88)	1 (1.79)	0 (0.00)	0.363
40–59	18 (15.93)	5 (8.93)	10 (20.41)	
60–69	34 (30.09)	17 (30.36)	16 (32.65)	
70–79	47 (41.59)	25 (44.64)	19 (38.78)	
80+	13 (11.50)	8 (14.29)	4 (8.16)	
*Gender*				
Male	60 (53.10)	32 (57.14)	25 (51.02)	0.561
Female	53 (46.90)	24 (42.86)	24 (48.98)	
*Race/ethnicity*				
Caucasian	98 (86.73)	50 (89.29)	42 (85.71)	0.114
Black	2 (1.77)	0 (0.00)	2 (4.08)	
Hispanic	7 (6.19)	4 (7.14)	2 (4.08)	
Asian	4 (3.54)	0 (0.00)	3 (6.12)	
Other	2 (1.77)	2 (3.57)	0 (0.00)	
*Tumor location*				
Head/uncinate	81 (71.68)	42 (75.00)	33 (67.35)	0.918
Neck	6 (5.31)	3 (5.36)	3 (6.12)	
Body/tail	26 (23.01)	11 (19.64)	13 (26.53)	
*Clinical stage at diagnosis*				
Stage IA	18 (15.93)	9 (16.07)	8 (16.33)	0.578
Stage IB	53 (46.90)	29 (51.79)	22 (44.90)	
Stage II	17 (15.04)	9 (16.07)	6 (12.24)	
Stage III	25 (22.12)	9 (16.07)	13 (26.53)	
*Resectability by Imaging Classification (NCCN)*				
Resectable	39 (34.51)	17 (30.36)	21 (42.86)	0.405
Borderline resectable	57 (50.44)	30 (53.57)	21 (42.86)	
Locally advanced	17 (15.04)	9 (16.07)	7 (14.29)	

ctDNA, circulating tumor DNA; n, number; NCCN, National Comprehensive Cancer Network. ^a^Percentages represent the proportion of patients within each category, calculated using the cohort size as the denominator. Minor discrepancies from 100% are due to missing data. ^b^Discrepancies between the total cohort and the number of individuals with detectable or nondetectable total ctDNA are due to some patients not undergoing NGS testing at diagnosis

Among 113 patients, 86.7% (98/113) received NAC, of whom 61.2% (60/98) underwent subsequent curative-intent resection. Mean NAC duration was 3.4 months (SD 2.1). Among NAC recipients, 59.1% (58/98) received FOLFIRINOX, 32.7% (32/98) gemcitabine/nab-paclitaxel, and 8.2% (8/98) mixed regimens due to toxicity-related changes. Nineteen patients did not complete planned NAC, most commonly due to disease progression or treatment-related toxicity (Supplemental Table 3). Among resected patients, 95.0% (57/60) achieved an R0 resection. Treatment pathways are summarized in Supplemental Fig. 2.

Supplemental Fig. 4 shows patient mutational profiles, including all mutations detected across any timepoint, with *TP53* (38/113, 33.6%) and *KRAS* (23/113, 20.4%) being most frequent. Detection rates by gene and timepoint are provided in Supplemental Table 4. Supplemental Table 5 details *KRAS* mutations detected per patient at any timepoint and includes available matched tumor *KRAS* molecular profiles for comparison in 39 patients.

### Circulating Tumor DNA Dynamics during Treatment Course

**Fig. 1:**
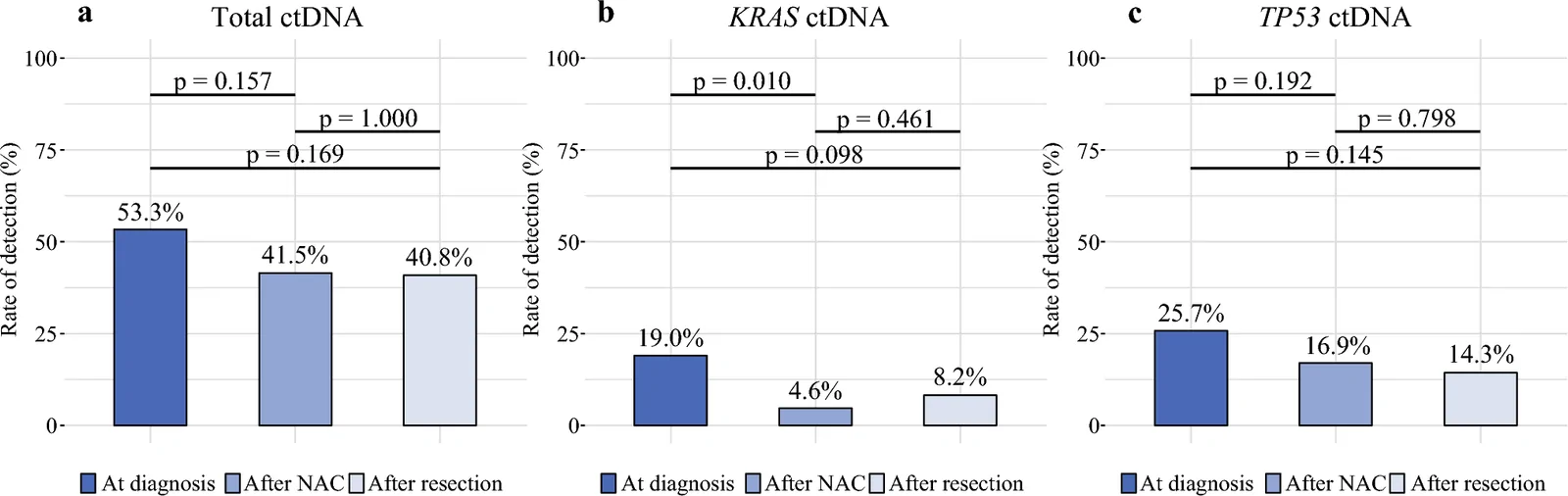
**a** Bar plot illustrates the rate of total ctDNA detection by NGS at three clinical timepoints: at diagnosis, after NAC, and after resection. **b** and **c** Detection rates of *KRAS*-specific and *TP53*-specific ctDNA, respectively

To assess ctDNA dynamics during treatment, we prospectively collected 219 blood samples from 113 patients across three timepoints (Supplemental Fig. 2). Total ctDNA detection by NGS was 53.3% (56/105) at diagnosis, 41.5% (27/65) post-NAC, and 40.8% (20/49) post-resection, with no significant differences across timepoints (Fig. 1a). Given their prevalence, *KRAS* and *TP53*-specific mutations were evaluated longitudinally. *KRAS* detection decreased significantly from diagnosis to post-NAC (19.0% [20/105] vs. 4.6% [3/65], *p* = 0.010), with no difference between post-NAC and postresection (4.6% [3/65] vs. 8.2% [4/49], *p* = 0.461), and a nonsignificant decrease between diagnosis and postresection (19% vs. 8.2%, *p* = 0.098; Fig. 1b). *TP53*-specific detection was 25.7% (27/105) at diagnosis, 16.9% (11/65) post-NAC, and 14.3% (7/49) postresection, with no significant differences (Fig. 1c). Although *SMAD4* and *CDKN2A* are important PDAC drivers, low baseline detection (1.9% [2/105] and 2.9% [3/105], respectively) precluded temporal analysis (Supplemental Table 5).^31^

### Circulating Tumor DNA Detection and Likelihood of Reaching Resection

**Fig. 2:**
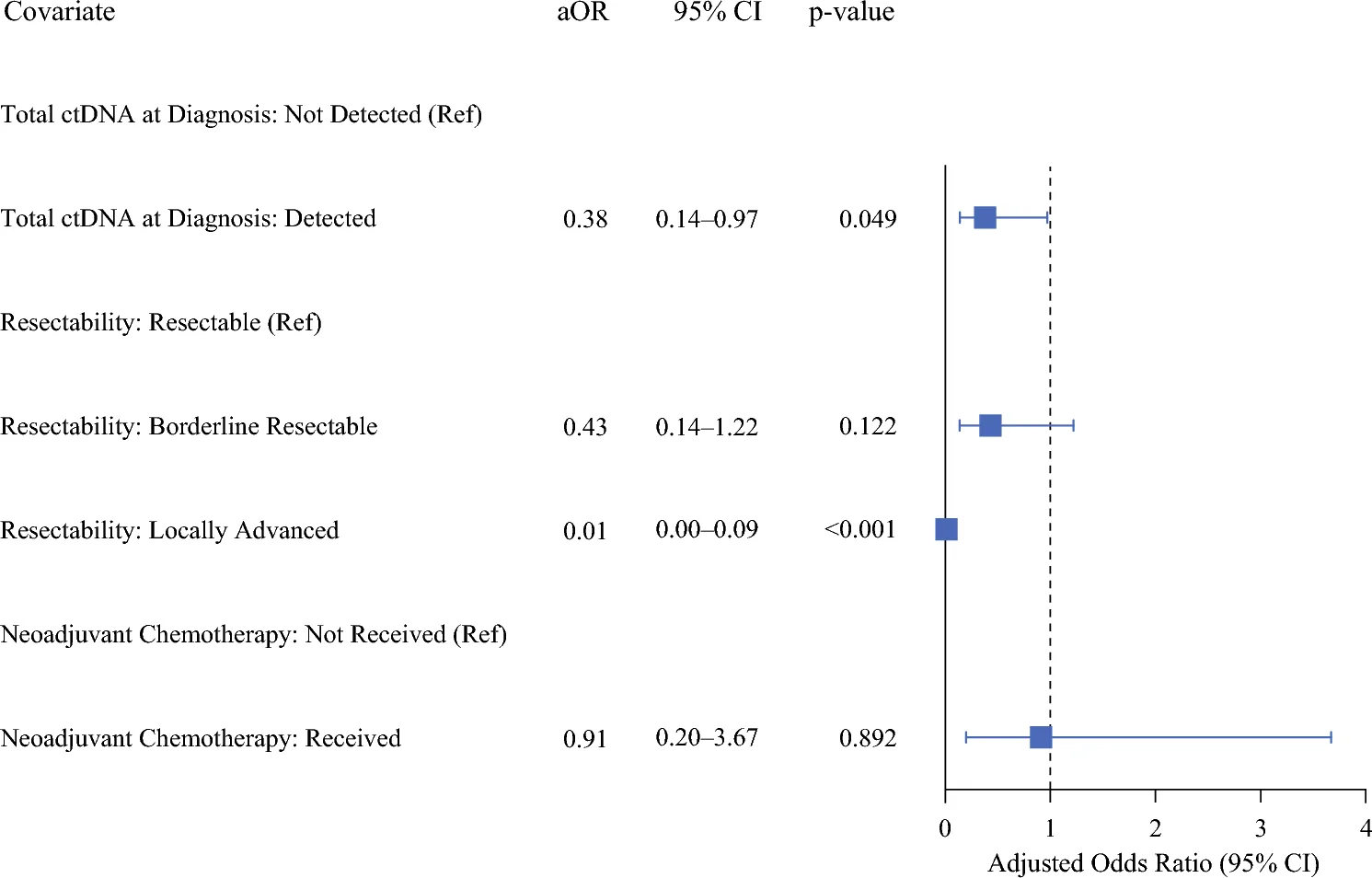
Forest plot of a multivariable logistic regression evaluating total ctDNA at diagnosis as a predictor of reaching resection

Overall, 38.1% (43/113) of patients did not undergo surgery. We next evaluated whether baseline total ctDNA detection predicted undergoing curative-intent resection, regardless of anatomic resectability. In multivariable logistic regression, total ctDNA detection at diagnosis independently predicted lower odds of receiving resection after adjusting for anatomic resectability and NAC receipt (aOR 0.38, 95% CI 0.14–0.97, *p* = 0.049; Fig. 2). Locally advanced status also predicted lower odds of resection compared with resectable patients (aOR 0.01, 95% CI 0.00–0.09, *p* < 0.001), whereas borderline-resectable status did not (aOR 0.43, 95% CI 0.14–1.22, *p* = 0.122). Given the low likelihood of resection among patients with locally advanced disease, we performed another regression excluding these patients, which again demonstrated that total ctDNA detection independently predicted lower odds of resection (aOR 0.31, 95% CI 0.10–0.84, *p* = 0.025).

We next examined reasons for not reaching resection by baseline total ctDNA status (Supplemental Table 6). In both ctDNA-detected and undetected groups, the most common reasons were metastatic progression, persistent unresectable anatomy, and death before surgical evaluation. A minority of patients received definitive chemoradiation based on treatment response or clinical judgment.

### Prognostic Significance of Circulating Tumor DNA Detection

**Fig. 3:**
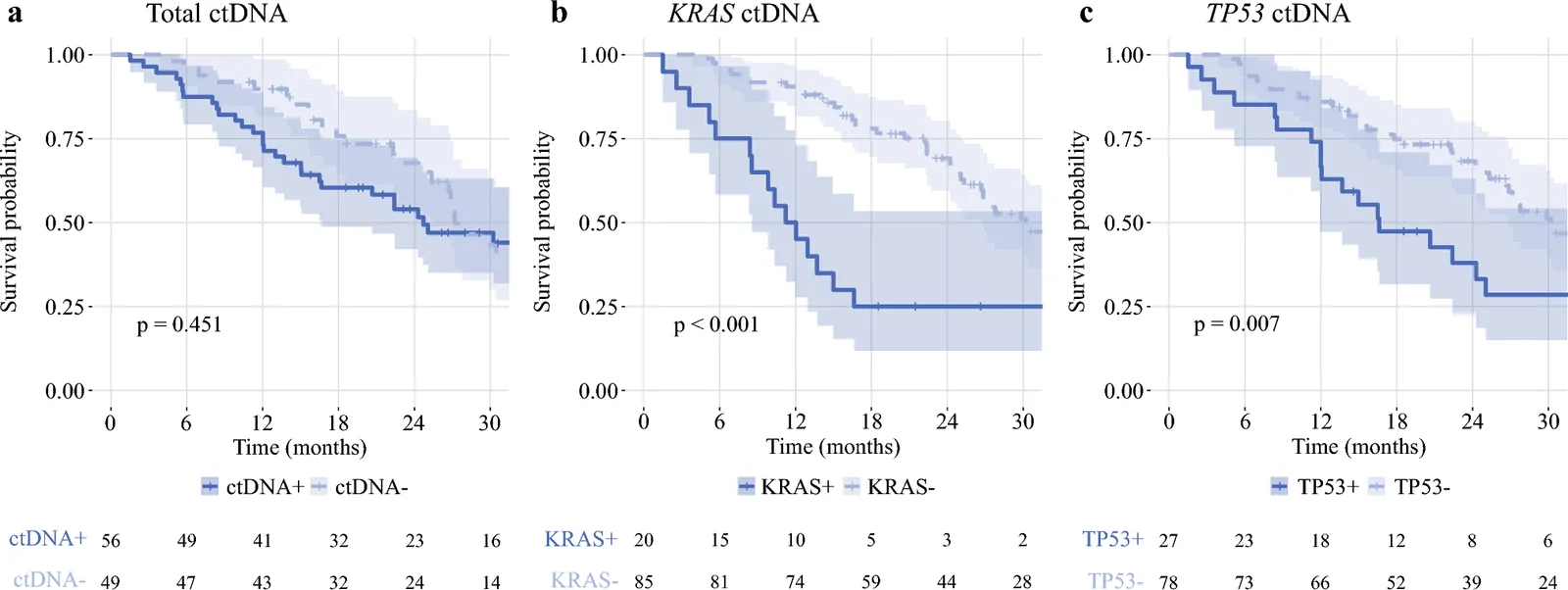
**a** Kaplan-Meier curve compares OS based on the presence or absence of total ctDNA by NGS at diagnosis. **b** and **c** depicts *KRAS*-specific ctDNA and *TP53*-specific ctDNA, respectively

As ctDNA was detected across timepoints and associated with resection, we next investigated its prognostic significance. Median follow-up was 22.1 months (interquartile range [IQR] 13.5–31.7). At diagnosis, total ctDNA detection was not associated with OS (mOS 24.7 vs. 27.2 months, *p* = 0.451; Fig. 3a). However, *KRAS*-specific (mOS 11.6 vs. 30.2 months, *p* < 0.001; Fig. 3b) and *TP53*-specific ctDNA were each associated with shorter OS (mOS 16.6 vs. 30.2 months, *p* = 0.007; Fig. 3c). Detection of total, *KRAS-*, or *TP53*-specific ctDNA post-NAC or post-resection was not associated with OS (Supplemental Fig. 5a-f).

**Fig. 4:**
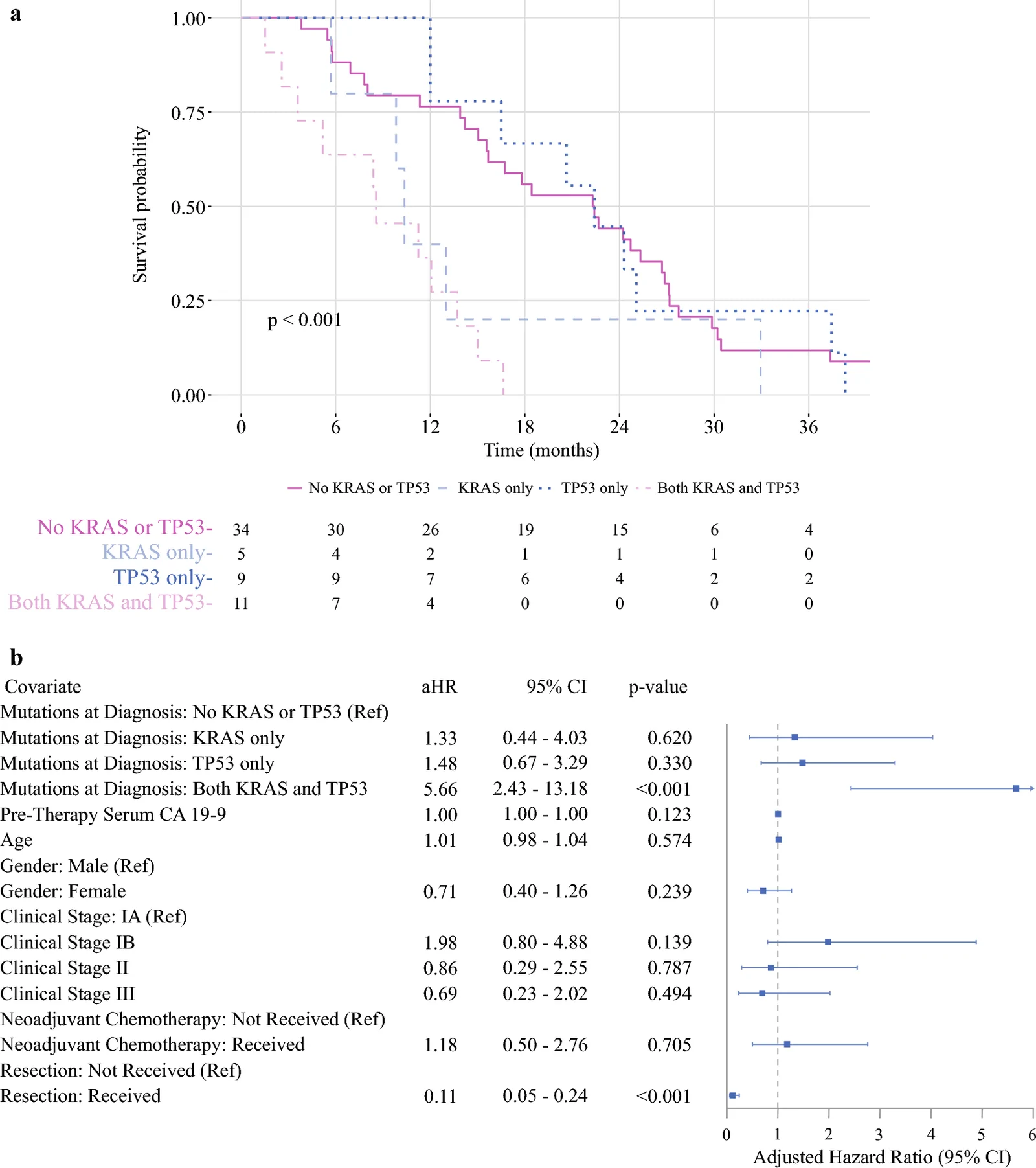
**a** Kaplan-Meier curve compares OS between four groups at diagnosis: no *KRAS* or *TP53* ctDNA, *KRAS* only, *TP53* only, and both *KRAS* and *TP53* ctDNA. **b** A forest plot of a multivariable Cox proportional hazards analysis evaluating *KRAS* and *TP53* ctDNA detection at diagnosis as predictors of OS

Given the association of *KRAS* and *TP53*-specific ctDNA with OS, we evaluated whether their co-occurrence, which is common in PDAC, could explain these findings.^32^ Patients were grouped by ctDNA status: no ctDNA mutations, *KRAS*-mutant ctDNA only, *TP53*-mutant ctDNA only, or both *KRAS* and *TP53*-mutant ctDNA. Survival differed significantly across groups (*p* < 0.001), with the shortest mOS in patients with co-occurring mutations (8.5 months), followed by *KRAS* only (10.3 months), *TP53* only (22.4 months), and neither mutation (22.4 months; Fig. 4a). In multivariable Cox analysis, co-occurrence independently predicted worse OS compared with no mutation (adjusted hazard ratio [aHR] 5.66, 95% CI 2.43–13.18, *p* < 0.001), whereas *KRAS*-only and TP53-only were not prognostic (Fig. 4b). Additionally, resection was associated with improved survival (aHR 0.11, 95% CI 0.05–0.24, *p* < 0.001), while age, gender, clinical stage, pretherapy CA 19-9, and receipt of NAC were not. Harrell’s C‑index was 0.80, indicating strong discriminatory performance in ranking patients by survival risk. To further explore longitudinal ctDNA dynamics, we examined the subgroup of patients with ctDNA assessed at all three timepoints (*n* = 39). In this small subgroup of patients, neither total ctDNA detection, mVAF, nor clearance was associated with survival.

### Prognostic Significance of Circulating Tumor DNA Detection Across Chemotherapy Regimens

Because ctDNA was prognostic at diagnosis, we assessed whether the chemotherapy regimen modified this effect by examining survival by ctDNA status in patients treated with FOLFIRINOX or gemcitabine, including those who received combination regimens in both analyses. Among FOLFIRINOX-treated patients, total ctDNA detection was not prognostic at any timepoint (Supplemental Fig. 6a-c). In contrast, *KRAS-* (mOS 13.9 vs. 38.3 months, *p* = 0.006) and *TP53*-specific ctDNA (mOS 16.6 vs 33.0 months, *p* = 0.015) were associated with worse survival at diagnosis but not at later timepoints (Supplemental Fig. 6d–i).

In gemcitabine-treated patients, total ctDNA was also not prognostic at any timepoint (Supplemental Fig. 7a-c). *KRAS* ctDNA (mOS 13.9 vs. 38.3 months, *p* = 0.006) was associated with worse survival at diagnosis but not later timepoints; post-NAC analysis was not possible as no patients had detectable *KRAS* ctDNA (Supplemental Fig. 7d-e). *TP53* ctDNA was not prognostic at any timepoint (all *p* > 0.05; Supplemental Fig. 7f-h).

### Prognostic Significance of Circulating Tumor DNA Mutational Burden

As ctDNA is a marker of disease burden, we next evaluated if treatment led to changes in quantitative mutational burden over time.^33^ No significant differences in mVAF were observed across treatment timepoints for total ctDNA (0.7% vs. 1.5% vs. 1%), *KRAS*-specific ctDNA (0.4% vs. 0.8% vs. 0.3%), or *TP53*-specific ctDNA (0.3% vs. 0.4% vs. 0.2%; all *p* > 0.05; Supplemental Fig. 8a-c).

**Fig. 5:**
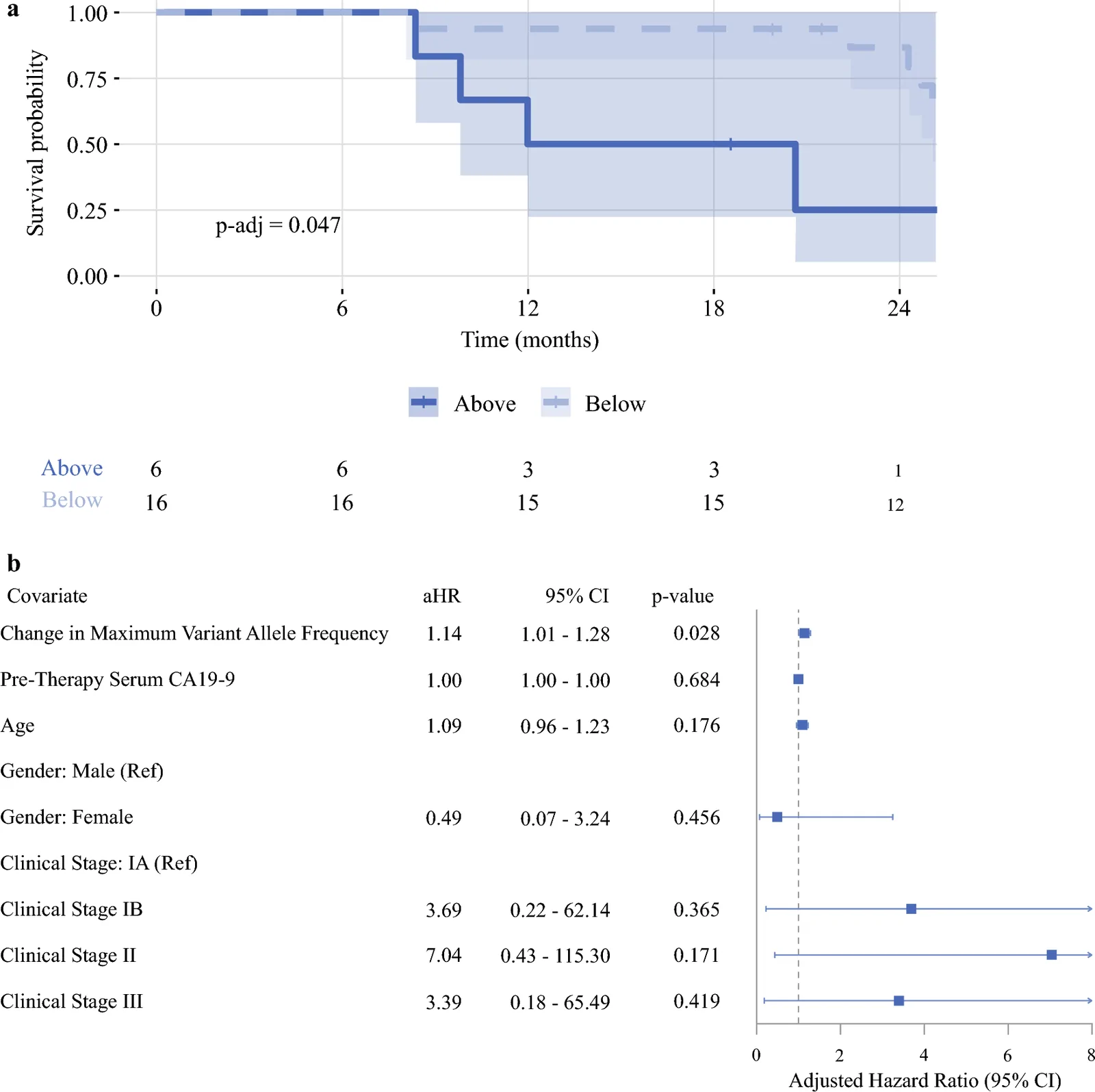
**a** Kaplan-Meier curve compares OS stratified by a 0.5% cutoff in total ctDNA mVAF change from diagnosis to post-NAC. Patients above the cutoff had an increase >0.5%, while those below the cutoff had a decrease, stable, or a smaller increase. **b** A forest plot of a multivariable Cox proportional hazards analysis evaluating total ctDNA mVAF change from diagnosis to post-NAC as a predictor of OS. *aHR* adjusted hazard ratio; *CA 19-9* carbohydrate antigen 19-9; *CI* confidence interval; *ctDNA* circulating tumor DNA; *KRAS* Kirsten rat sarcoma virus gene; *mVAF* maximum variant allele frequency; *NAC* neoadjuvant chemotherapy; *NGS* next-generation sequencing; *OS* overall survival; *TP53* tumor protein p53

We next assessed whether changes in total ctDNA mVAF from diagnosis to post-NAC were associated with survival among patients with detectable ctDNA at both timepoints. Although median cohort-level mVAF did not change over treatment, patients with stable or decreased mVAF had longer OS than those with an mVAF increase >0.5% (mOS NR vs. 16.3 months, p-adj = 0.047; Fig. 5a). When modeling mVAF change continuously in multivariable Cox analysis, increasing mVAF independently predicted shorter OS (aHR 1.14, 95% CI 1.01–1.28, *p* = 0.028), whereas age, gender, clinical stage, and pre-therapy serum CA 19-9 were not significant (Fig. 5b). Harrell’s C‑index was 0.73, indicating good discriminatory performance in ranking patients by survival risk.

## Discussion

In this study, we assessed the clinical utility of ctDNA in PDAC using NGS at diagnosis, post-NAC, and post-resection, extending our prior findings that *KRAS*-specific ctDNA predicts PFS.^23^ Our tumor-agnostic NGS panel showed a reduction in *KRAS*-specific ctDNA following NAC. Also, baseline total ctDNA predicted lower odds of resection. Additionally, co-occurrence of *KRAS* and *TP53* mutations predicted shorter OS. Finally, increases in total ctDNA mutational burden during treatment predicted poor prognosis. These results support NGS-based ctDNA monitoring as a clinically actionable biomarker of treatment response and long-term outcomes in localized PDAC.

Our tumor-agnostic NGS approach enabled simultaneous profiling of multiple cancer-relevant genes and quantification of total mutational burden via mVAF, aiding in the identification of potentially actionable alterations for longitudinal monitoring in clinical trials.^34^ Importantly, this tumor-agnostic approach was selected for scalability and real‑world feasibility, as tumor‑agnostic assays offer faster turnaround times and require less infrastructure than tumor‑informed platforms, which depend on tissue availability and assay customization. These are important limitations in PDAC given frequent tissue scarcity, posttreatment low cellularity, and evolving driver mutations under therapeutic pressure.^35^ Using this approach, total ctDNA was detected in 53.3% of patients at diagnosis, consistent with prior reports (30–60%).^17,23,36–39^

Our post-NAC and postresection detection rates were comparable to studies conducting longitudinal monitoring in the neoadjuvant setting.^38,39^ However, our observed total ctDNA clearance (53.3% at diagnosis to 41.5% post-NAC) differed from Cecchini et al., who reported a decrease from 73% at diagnosis to 18% post-NAC.^40^ A contributing factor is likely due to their use of a tumor-informed assay, which tracks only variants identified in surgically resected tumor tissue without focusing on low‑frequency or spatially distinct resistant subclones. As a result, post-treatment ctDNA detection may differ from alterations present at diagnosis due to therapeutic pressure. In contrast, our tumor‑agnostic approach assessed a broader mutational landscape and may therefore demonstrate greater persistence of total ctDNA. Importantly, persistent ctDNA likely reflects the aggressive, therapy‑resistant biology of PDAC rather than assay limitations alone.

While total ctDNA detection remained stable across treatment, *KRAS*-specific ctDNA declined following NAC, suggesting dynamic treatment responsiveness consistent with previous studies.^15,41^ The stability of total ctDNA despite declining *KRAS* may reflect treatment-induced shifts in the tumor’s molecular landscape, with other mutations masking mutation-specific dynamics.^42–44^ In contrast, *TP53* detection showed a non-significant decrease after NAC, suggesting lower treatment responsiveness. Together, these findings highlight the value of integrating mutation-specific ctDNA, particularly *KRAS*, over aggregate ctDNA alone, and suggest that failure of *KRAS* ctDNA to decline with therapy may identify patients at higher risk of early progression who may derive limited benefit from surgery.^45^

Additionally, *KRAS*-specific ctDNA detection increased postoperatively compared with post-NAC, though not significantly. This may reflect residual micrometastatic disease, as NAC can suppress occult deposits, while unresected micrometastases may drive postoperative ctDNA.^46,47^ Prior studies, however, report significant declines in *KRAS* ctDNA postresection due to reduced tumor burden.^17,38^ Overall, these findings suggest micrometastatic disease may contribute to postoperative *KRAS* detection, although our data cannot confirm this.

Despite these insights, *KRAS* (4.6%–19%) and *TP53*-specific ctDNA (14.3%–25.7%) detection rates were relatively low across timepoints, despite these mutations occurring in approximately 95% and 75% of PDACs, respectively.^48^ Our *KRAS* detection rate was lower than Macgregor-Das et al. (36.5%), likely reflecting our focus on localized PDAC, where lower tumor burden limits ctDNA shedding, and is consistent with other reports in this setting.^23,49,50^ Importantly, our goal was to evaluate ctDNA as a prognostic biomarker rather than to identify driver mutations. Detection depends on sufficient ctDNA burden, and we are exploring its utility for risk stratification.

Because not all patients proceeded to surgery, we evaluated whether baseline total ctDNA predicted failure to reach resection. Resection rates decreased stepwise by resectability: 82.1% in resectable, 64.9% in borderline-resectable, and 5.9% in locally advanced patients, similar to those reported by Brown et al. (77.4%, 60.6%, and 22.2%, respectively).^51^ Detectable total ctDNA at diagnosis was associated with lower odds of surgery, consistent with its links to worse survival, recurrence, occult metastases, and poorer NAC response.^23,45,52–54^ This predictive value persisted after adjusting for anatomic resectability. Given prior evidence that anatomic resectability and other preoperative factors poorly predict R0 resection, total ctDNA may provide prognostic information beyond anatomy, and incorporating it alongside resectability status may help identify patients unlikely to reach surgery and inform strategies, such as extended NAC.^55,56^

Beyond predicting surgical candidacy, we evaluated the prognostic value of ctDNA. While total ctDNA was not associated with survival, co-occurring *KRAS* and *TP53* ctDNA at diagnosis independently predicted adverse outcomes, highlighting its value as a prognostic biomarker in localized PDAC. Importantly, detection of *KRAS*‑ or *TP53*‑specific ctDNA should not be interpreted as reflecting primary tumor mutational status, as these alterations are nearly ubiquitous in PDAC and their absence in plasma does not imply absence in tumor tissue. Moreover, prior studies have shown that ctDNA levels do not necessarily correlate with primary tumor volume.^57^ Rather, ctDNA detectability in localized disease likely reflects systemic disease burden and aggressive biology, including occult micrometastatic spread and extensive nodal involvement. In this context, detectable *KRAS*‑ or *TP53*‑specific ctDNA likely represents increased tumor DNA shedding and is consistent with the shorter survival observed. These findings are consistent with the oncogenic roles of *KRAS* and *TP53*.^58–60^ Our prior NGS study in a smaller cohort showed *KRAS*-only and *KRAS*-*TP53* co-detection predicted worse PFS.^23^ Our current findings suggest that co-occurring *KRAS*–*TP53* alterations may be most predictive of shorter OS.

These data support *KRAS-* and *TP53*-specific ctDNA as biomarkers of disease aggressiveness, with potential to refine risk stratification and guide individualized treatment, including therapy escalation or de-escalation.^61^ This is increasingly relevant with the development of *KRAS*-targeted therapies, including pan-*KRAS* inhibitors, and emerging *TP53*-targeted approaches in PDAC.^62–66^ Detection of these alterations could help identify high-risk patients who may benefit from these personalized approaches. Notably, CA 19-9 did not independently predict survival. Although lower pre-operative CA 19-9 is generally associated with improved OS, its prognostic value appears limited when accounting for clinical and molecular factors, emphasizing the added value of ctDNA.^67^

We additionally analyzed whether chemotherapy regimen modified the prognostic impact of mutation-specific ctDNA. In FOLFIRINOX-treated patients, *KRAS* and *TP53*-specific ctDNA at diagnosis were associated with shorter survival, whereas in gemcitabine-treated patients, only *KRAS* ctDNA was prognostic. Together, these data suggest that mutation-specific DNA may non-invasively identify patients at higher risk of shorter survival despite chemotherapy.

Although detection declined after NAC, *KRAS*-specific mVAF remained stable over timepoints, as did total and *TP53*-specific mVAF, suggesting that patients with persistent detection maintained a steady mutational burden. Even without cohort-level changes in mVAF, increases in total ctDNA mVAF over treatment predicted shorter OS. Additionally, stratification by a cutoff revealed that larger increases were associated with worse survival. This suggests that rising mutational burden over treatment may reflect therapy resistance and subclinical disease progression, including expansion of micrometastatic clones despite ongoing treatment. These findings support the potential utility of monitoring total ctDNA mutational burden to identify high-risk patients after NAC who may benefit from closer surveillance or alternative therapies.

### Limitations

This study has several limitations. Despite being a large prospective cohort, sample size limited subgroup analyses. Few patients achieved ctDNA clearance, restricting assessment of its prognostic value. Also, follow-up time was limited for long-term survival. Additionally, ctDNA was not available at all timepoints for all patients, reflecting real-world challenges of prospective longitudinal sampling in a large cohort and reducing statistical power; nevertheless, 219 blood samples were collected, yielding a robust prospective dataset without the selection bias inherent to retrospective studies. Also, samples were not collected during NAC, limiting evaluation of early treatment-response dynamics. Lastly, plasma NGS detection reflects both assay sensitivity and disease burden; while reliable in higher-burden disease, low‑burden cases may fall below its limit of detection, potentially missing low-frequency variants and contributing to modest *KRAS* and *TP53* ctDNA detection, particularly relative to higher‑sensitivity approaches, such as digital droplet PCR. Nonetheless, our NGS approach provided broader coverage and scalability. Ongoing recruitment and longer follow-up will improve future analyses, especially in patients achieving ctDNA clearance.

## Conclusions

In this prospective cohort of localized PDAC patients undergoing NAC and resection, NGS-detected ctDNA showed prognostic value. Total ctDNA detection remained stable, while *KRAS*-specific ctDNA declined after NAC, indicating potential as a treatment-response marker. Baseline total ctDNA independently predicted lower likelihood of resection, and co-occurring *KRAS* and *TP53*-specific ctDNA predicted poor outcomes. Increases in total ctDNA mVAF over treatment also predicted shorter OS. These results support mutation-specific ctDNA analysis for risk stratification and treatment monitoring, warranting validation in larger cohorts with longer follow-up.

## Supplementary Information

Below is the link to the electronic supplementary material.

Supplementary file1 (DOCX 3127 kb)

## Data Availability

The data that support the findings of this study are available from the corresponding author upon reasonable request.
