# Supplementary material for: KRAS and TP53 Circulating Tumor DNA are Prognostic in Localized Pancreatic Cancer
Source: Ann Surg Oncol. 2026 Aug 26;33(12):10138–49. doi: 10.1245/s10434-026-20447-0 (PMC13675306; doi:10.1245/s10434-026-20447-0)
Supplement: Supplementary file 1 [file 10434_2026_20447_MOESM1_ESM.docx]

**Supplemental Information**

Supplemental Information Legends

Supplemental Table 1: Genes included in the Tempus X|F gene panel [1]

Supplemental Table 2: Genes included in the PredicineCARE^TM^ gene panel [2]

Supplemental Table 3: Reasons for early interruption or discontinuation of neoadjuvant chemotherapy

Supplemental Table 4: Detection rates of ctDNA gene alterations across treatment timepoints

Supplemental Table 5: Patient‑level ctDNA and tissue mutational profiles detected during the study period

Supplemental Table 6: Reasons for not undergoing surgical resection among study patients

Supplemental Figure 1: Flow diagram depicting the cohort formation process, outlining the exclusions and inclusion criteria for the study participants

Supplemental Figure 2: Sankey diagram showing patient enrollment, sample collection, treatment pathway, and ctDNA detection

Supplemental Figure 3: Kaplan-Meier curve depicting overall survival by NGS assay: Tempus vs Predicine

Supplemental Figure 4: Oncoplot illustrating mutations identified by NGS across all patients in the cohort. Filled boxes indicate the presence of specific mutations detected in each patient at any timepoint during the study.

Supplemental Figure 5a-f: Supplemental Figure 5a-b show Kaplan-Meier curves comparing OS based on the presence or absence of detectable total ctDNA by NGS after NAC and after resection. Supplemental Figures 5c–d present Kaplan–Meier curves comparing overall survival by *KRAS*-specific ctDNA detection at the same timepoints, while Supplemental Figure 5e–f display analogous curves stratified by *TP53*-specific ctDNA detection.

Supplemental Figure 6a-i: Supplemental Figure 6a-c show Kaplan-Meier curves comparing OS based on the presence or absence of detectable total ctDNA by NGS at diagnosis, after NAC, and after resection for patients receiving neoadjuvant FOLFIRINOX. Supplemental Figure 6d–f present Kaplan–Meier curves comparing overall survival by *KRAS*-specific ctDNA detection at the same timepoints, while Supplemental Figure 6g–i display analogous curves stratified by *TP53*-specific ctDNA detection.

Supplemental Figure 7a-i: Supplemental Figure 7a-c show Kaplan-Meier curves comparing OS based on the presence or absence of detectable total ctDNA by NGS at diagnosis, after NAC, and after resection for patients receiving neoadjuvant gemcitabine. Supplemental Figure 7d–e present Kaplan–Meier curves comparing overall survival by *KRAS*-specific ctDNA detection at the same timepoints, while Supplemental Figure 7f–h display analogous curves stratified by *TP53*-specific ctDNA detection.

Supplemental Table 1: Genes included in the Tempus X|F gene panel [1]

| AKT1 | FGFR3 | NOTCH1 |
| --- | --- | --- |
| AKT2 | FHFR4 | NPM1 |
| ALK | FLT3 | NRAS |
| APC | FOXL2 | NTRK1 |
| AR | GATA3 | PALB2 |
| ARAF | GNA11 | PBRM1 |
| ARID1A | GNAQ | PDCD1LG2 |
| ATM | GNAS | PDGFRA |
| ATR | HNF1A | PDGFRB |
| B2M | HRAS | PIK3CA |
| BAP1 | IDH1 | PIK3R1 |
| BRAF | IDH2 | PMS2 |
| BRCA1 | JAK1 | PTCH1 |
| BRCA2 | JAK2 | PTEN |
| BTK | JAK3 | PTPN11 |
| CCND1 | KDR | RAD51C |
| CCND2 | KEAP1 | RAF1 |
| CCND3 | KIT | RB1 |
| CCNE1 | KMT2A | RET |
| CD274 (PD-L1) | KRAS | RHEB |
| CDH1 | MAP2K1 | RHOA |
| CDK4 | MAP2K2 | RIT1 |
| CDK6 | MAPK1 | RNF43 |
| CDKN2A | MET | ROS1 |
| CTNNB1 | MLH1 | SDHA |
| DDR2 | MPL | SMAD4 |
| DPYD | MSH2 | SMO |
| EGFR | MSH3 | SPOP |
| ERBB2 (HER2) | MSH6 | STK11 |
| ERRFI1 | MTOR | TERT |
| ESR1 | MYC | TP53 |
| EZH2 | MYCN | TSC1 |
| FBXW7 | NF1 | TSC2 |
| FGFR1 | NF2 | UGT1A1 |
| FGFR2 | NFE2L2 | VHL |

Supplemental Table 2: Genes included in the PredicineCARE^TM^ gene panel [2]

| ABRAXAS1 | AKT1 | AKT2 |
| --- | --- | --- |
| AKT3 | ALK | APC |
| AR | ARAF | ARID1A |
| ATM | ATRX | BAP1 |
| BARD1 | BCL2 | BRAF |
| BRCA1 | BRCA2 | BRIP1 |
| BTK | CCND1 | CCND2 |
| CCND3 | CCNE1 | CCNE2 |
| CD274 | CD74 (PD-L1) | CDH1 |
| CDK12 | CDK2 | CDK4 |
| CDK6 | CDKN2A | CHEK1 |
| CHEK2 | CTNNB1 | CXCR4 |
| CYP2C19 | CYP2D6 | CYP3A4 |
| DAXX | DDR2 | DPYD |
| E2F1 | EGFR | EPCAM |
| ERBB2 (HER2) | ERBB3 | ERCC1 |
| ESR1 | EZH2 | FANCA |
| FANCC | FANCF | FANCG |
| FANCL | FAT1 | FBXW7 |
| FEN1 | FGFR1 | FGFR2 |
| FGFR3 | FGFR4 | FLT3 |
| FOXA1 | FOXL2 | FZR1 |
| GEN1 | GNA11 | GNAQ |
| GNAS | GSTP1 | HNF1A |
| HOXB13 | HRAS | IDH1 |
| IDH2 | JAK2 | JAK3 |
| KDM6A | KIT | KMT2C |
| KMT2D (MLL2) | KRAS | MAP2K1 (MEK1) |
| MAP2K2 (MEK2) | MAPK1 | MAPK3 |
| MDM2 | MET | MLH1 |
| MPL | MRE11 | MSH2 |
| MSH6 | MTHFR | MTOR |
| MYC | MYCN | MYD88 |
| NBN | NF1 | NFE2L2 |
| NOTCH1 | NPM1 | NRAS |
| NTRK1 | NTRK2 | NTRK3 |
| PALB2 | PDCD1LG2 (PD-L2) | PDGFRA |
| PIK3CA | PIK3CB | PIK3R1 |
| PLCG2 | PMS2 | POLD1 |
| POLE | PPP2R1A | PRKACA |
| PRKD1 | PTEN | PTPN11 |
| RAD50 | RAD51 | RAD51B |
| RAD51C | RAD51D | RAD52 |
| RAF1 | RB1 | RET |
| RHEB | RHOA | RIT1 |
| RNF43 | ROS1 | SDHB |
| SMAD4 | SMO | SPOP |
| STAG2 | STK11 | TERT promoter |
| TMPRSS2 | TP53 | TSC1 |
| TSC2 | UGT1A1 | VHL |
| XPC | XRCC1 |  |

Supplemental Table 3: Reasons for early interruption or discontinuation of neoadjuvant chemotherapy

| **ID** | **Reason for NAC interruption or discontinuation** |
| --- | --- |
| 1 | Treatment discontinued after 4 of 8 planned cycles due to development of metastatic disease identified on interval CT imaging. |
| 3 | Cycle 3 was delayed by 3 weeks and Cycle 4 by 1 week due to thrombocytopenia. |
| 5 | No further chemotherapy was administered after Cycle 3 due to hospitalization and surgical intervention for superior mesenteric artery and gastroduodenal hemorrhage secondary to metastatic retroperitoneal lymph node disease with duodenal erosion by the primary tumor, requiring transfusions. Treatment was further limited by declining performance status and suspected disease progression to the liver. |
| 14 | Initially treated with gemcitabine/abraxane (days 1 and 15 of a 28‑day cycle); possible disease progression after 3 cycles prompted transition to modified FOLFIRINOX (every 14 days), with a total of 4 cycles administered. Treatment was complicated by fatigue and diarrhea, necessitating 20% dose reductions beginning with Cycle 3. Cycle 2 was delayed by 5 days and Cycle 3 by 2 weeks. No dose reductions were required during gemcitabine/abraxane therapy. |
| 17 | Completed 5 cycles of modified FOLFIRINOX with poor tolerability and no delays. Subsequent imaging demonstrated hepatic progression, after which the patient declined further treatment. |
| 24 | Given a decrease in CA 19‑9 levels, the decision was made to pursue surgical resection after 2 cycles rather than the initially planned 4 cycles of treatment. |
| 30 | Imaging demonstrated disease progression after 3 cycles of gemcitabine/abraxane; chemotherapy was continued without surgery, and the regimen was changed to modified FOLFOX6 (every 14 days), with 6 cycles administered. |
| 32 | Treatment initiation was delayed due to elevated liver function tests and the need for biliary stent placement. Cycle 8 chemotherapy was omitted in favor of radiation therapy. |
| 33 | Cycle 2 Day 15 of gemcitabine/abraxane (total of 4 neoadjuvant cycles) was delayed by 1 week due to fatigue. Chemotherapy was discontinued after 4 cycles following imaging that revealed diffuse hepatic metastases, progression of the primary pancreatic mass, and regional progression in peripancreatic lymph nodes. |
| 36 | Cycle 2 Day 1 of gemcitabine/abraxane was delayed by 1 week due to recent hospitalization for altered mental status. Cycle 5 Day 15 was omitted due to hospitalization for liver abscess, and Cycle 6 Day 15 was not administered due to hospitalization for fever. |
| 44 | Disease progression to the liver prompted pursuit of additional opinions; no further treatment was administered, and the patient subsequently died. |
| 54 | During Cycle 3, the patient developed dyspnea following irinotecan infusion and was admitted for pancreatitis and cholecystitis, thought to be treatment‑related. |
| 72 | Treatment was discontinued following detection of metastatic disease. |
| 76 | Hospitalization for sepsis occurred after Cycle 7, followed by rehabilitation. |
| 86 | Patient died after completion of 2 cycles of therapy. |
| 100 | Patient was hospitalized with sepsis and died. Chemotherapy was switched to gemcitabine/abraxane due to thrombocytopenia despite platelet transfusions and dose reductions. |
| 107 | Completed 8 cycles of FOLFIRINOX with poor tolerability and limited response; treatment was subsequently switched to gemcitabine/abraxane. |
| 108 | Treatment discontinued due to disease progression after 2 cycles. |
| 110 | Cycle 6 was delayed by 1 week due to uncontrolled atrial fibrillation with rapid ventricular response. |

^a^Patient identifiers correspond to those shown in the oncoplot in Supplemental Figure 4.

Abbreviations: ID, identification

Supplemental Table 4: Detection rates of ctDNA gene alterations across treatment timepoints

| Gene | Detection rates at timepoints, n (%) | | |
| --- | --- | --- | --- |
|  | At diagnosis, N = 105 | After NAC, N = 65 | After resection, N = 49 |
| AR | 1 (0.95) | 1 (1.54) | 1 (2.04) |
| ARID1A | 2 (1.90) | 0 (0.00) | 0 (0.00) |
| ATM | 4 (3.81) | 1 (1.54) | 1 (2.04) |
| ATRX | 3 (2.86) | 3 (4.62) | 0 (0.00) |
| BAP1 | 0 (0.00) | 1 (1.54) | 0 (0.00) |
| BRCA1 | 1 (0.95) | 1 (1.54) | 1 (2.04) |
| BRCA2 | 2 (1.90) | 1 (1.54) | 1 (2.04) |
| CDK4 | 1 (0.95) | 0 (0.00) | 0 (0.00) |
| CDKN2A | 3 (2.86) | 0 (0.00) | 1 (2.04) |
| CDKN2B | 1 (0.95) | 0 (0.00) | 0 (0.00) |
| CHEK2 | 1 (0.95) | 0 (0.00) | 0 (0.00) |
| DNMT3A | 1 (0.95) | 0 (0.00) | 0 (0.00) |
| EGFR | 2 (1.90) | 1 (1.54) | 0 (0.00) |
| EZH2 | 3 (2.86) | 0 (0.00) | 1 (2.04) |
| FBXW7 | 0 (0.00) | 0 (0.00) | 1 (2.04) |
| FGFR1 | 1 (0.95) | 1 (1.54) | 0 (0.00) |
| FGFR2 | 0 (0.00) | 1 (1.54) | 0 (0.00) |
| FGFR3 | 1 (0.95) | 0 (0.00) | 0 (0.00) |
| GNA11 | 1 (0.95) | 1 (1.54) | 0 (0.00) |
| GNAS | 0 (0.00) | 1 (1.54) | 1 (2.04) |
| IDH1 | 1 (0.95) | 0 (0.00) | 0 (0.00) |
| IDH2 | 1 (0.95) | 0 (0.00) | 0 (0.00) |
| JAK3 | 1 (0.95) | 1 (1.54) | 1 (2.04) |
| KIT | 1 (0.95) | 1 (1.54) | 0 (0.00) |
| KRAS | 20 (19.05) | 3 (4.62) | 4 (8.16) |
| MLH1 | 2 (1.90) | 1 (1.54) | 1 (2.04) |
| MYC | 2 (1.90) | 0 (0.00) | 0 (0.00) |
| MYCN | 3 (2.86) | 4 (6.15) | 2 (4.08) |
| MYD88 | 2 (1.90) | 0 (0.00) | 0 (0.00) |
| NF1 | 1 (0.95) | 1 (1.54) | 1 (2.04) |
| NRAS | 1 (0.95) | 1 (1.54) | 0 (0.00) |
| PIK3CA | 3 (2.86) | 2 (3.08) | 0 (0.00) |
| PPP2R2A | 3 (2.86) | 4 (6.15) | 1 (2.04) |
| PTEN | 2 (1.90) | 4 (6.15) | 1 (2.04) |
| PTPN11 | 1 (0.95) | 0 (0.00) | 0 (0.00) |
| RB1 | 4 (3.81) | 3 (4.62) | 0 (0.00) |
| RNF43 | 1 (0.95) | 0 (0.00) | 0 (0.00) |
| SMAD4 | 2 (1.90) | 0 (0.00) | 0 (0.00) |
| SMARCA4 | 1 (0.95) | 0 (0.00) | 0 (0.00) |
| TP53 | 27 (25.71) | 11 (16.92) | 7 (14.29) |
| TSC1 | 1 (0.95) | 1 (1.54) | 0 (0.00) |
| VHL | 1 (0.95) | 0 (0.00) | 0 (0.00) |

Abbreviations: ctDNA, circulating tumor DNA; NAC, neoadjuvant chemotherapy

Supplemental Table 5: Patient‑level ctDNA and tissue *KRAS* mutational profiles detected during the study period

| **PID^a^** | ***KRAS* Mutational Profile** | |
| --- | --- | --- |
|  | **ctDNA** | **Tissue** |
| 1 | Not detected^b^ | G12V |
| 2 | Not detected | No tissue^c^ |
| 3 | Not detected | No tissue |
| 4 | Not detected | G12D |
| 5 | G12D | No tissue |
| 6 | G12R | No tissue |
| 7 | Not detected | No tissue |
| 8 | Not detected | G12R |
| 9 | Not detected | No tissue |
| 10 | Not detected | No tissue |
| 11 | Not detected | G12D |
| 12 | Not detected | No tissue |
| 13 | Not detected | No tissue |
| 14 | Not detected | No tissue |
| 15 | Not detected | G12R |
| 16 | Not detected | G12D |
| 17 | G12R | No tissue |
| 18 | Not detected | No tissue |
| 19 | Not detected | G12R |
| 20 | Not detected | No tissue |
| 21 | G12D | No tissue |
| 22 | Not detected | No tissue |
| 23 | Not detected | No tissue |
| 24 | G12D | G12D |
| 25 | Not detected | G12R |
| 26 | Not detected | G12D |
| 27 | Not detected | No tissue |
| 28 | Not detected | No tissue |
| 29 | Not detected | G12D |
| 30 | G12V | G12V |
| 31 | Not detected | No tissue |
| 32 | Not detected | No tissue |
| 33 | G12V | G12V |
| 34 | Not detected | G12R |
| 35 | Not detected | No tissue |
| 36 | Not detected | No tissue |
| 37 | Not detected | No tissue |
| 38 | Not detected | G12D |
| 39 | Not detected | No tissue |
| 40 | Not detected | G12D |
| 41 | G12R | No tissue |
| 42 | Not detected | G12V |
| 43 | Not detected | No tissue |
| 44 | Not detected | G12D |
| 45 | Not detected | No tissue |
| 46 | Not detected | No tissue |
| 47 | Not detected | No tissue |
| 48 | Not detected | No tissue |
| 49 | Not detected | No tissue |
| 50 | Not detected | No tissue |
| 51 | Not detected | G12R |
| 52 | G12V | G12V |
| 53 | Not detected | No tissue |
| 54 | Not detected | No tissue |
| 55 | G12D | No tissue |
| 56 | Not detected | G12R |
| 57 | Not detected | No tissue |
| 58 | Not detected | G12D |
| 59 | Not detected | No tissue |
| 60 | G12V | No tissue |
| 61 | Q61K | G12V |
| 62 | Not detected | No tissue |
| 63 | Not detected | No tissue |
| 64 | Not detected | No tissue |
| 65 | Not detected | Not detected |
| 66 | Not detected | No tissue |
| 67 | Not detected | No tissue |
| 68 | Not detected | Not detected |
| 69 | G12V | No tissue |
| 70 | Not detected | No tissue |
| 71 | Not detected | No tissue |
| 72 | G12D | G12D |
| 73 | Not detected | No tissue |
| 74 | Not detected | No tissue |
| 75 | Not detected | No tissue |
| 76 | Not detected | G12R |
| 77 | Not detected | No tissue |
| 78 | Not detected | No tissue |
| 79 | V14I | Not detected |
| 80 | Not detected | No tissue |
| 81 | G12R | No tissue |
| 82 | K117N | G12D |
| 83 | Not detected | No tissue |
| 84 | Not detected | No tissue |
| 85 | Not detected | No tissue |
| 86 | G12D | No tissue |
| 87 | Not detected | No tissue |
| 88 | Not detected | G12R |
| 89 | Not detected | No tissue |
| 90 | G12D | No tissue |
| 91 | Not detected | G12V |
| 92 | Not detected | G12D |
| 93 | G12D | G12D |
| 94 | Not detected | No tissue |
| 95 | Not detected | G12D |
| 96 | Not detected | No tissue |
| 97 | Not detected | No tissue |
| 98 | Not detected | No tissue |
| 99 | Not detected | No tissue |
| 100 | Not detected | G12D |
| 101 | Not detected | No tissue |
| 102 | K5Q | No tissue |
| 103 | G12R | No tissue |
| 104 | Not detected | G12D |
| 105 | Q61H | p.Q61H |
| 106 | Not detected | No tissue |
| 107 | Not detected | No tissue |
| 108 | Not detected | G12D |
| 109 | Not detected | No tissue |
| 110 | Not detected | No tissue |
| 111 | Not detected | G12D |
| 112 | Not detected | No tissue |
| 113 | Not detected | Not detected |

^a^Patient identifiers correspond to those shown in the oncoplot in Supplemental Figure 4.

^b^No mutations were detected in plasma.

^c^No tissue was collected for this patient.

Abbreviations: ID, identification

Supplemental Table 6: Reasons for not undergoing surgical resection among patients treated with NAC

| **Patient ID*** | **Total ctDNA Detection at Diagnosis** | **Resectability Status at Diagnosis** | **Received NAC** | **Reason for Not Reaching Surgery** |
| --- | --- | --- | --- | --- |
| 1 | Detected | Borderline Resectable | Yes | Metastatic progression during NAC |
| 3 | Detected | Locally Advanced | Yes | Death prior to surgical planning |
| 5 | Detected | Borderline Resectable | Yes | Metastatic progression; decline in performance status |
| 6 | Detected | Locally Advanced | Yes | Metastatic progression |
| 8 | Not Performed | Borderline Resectable | Yes | Metastatic disease discovered intraoperatively |
| 10 | Not Detected | Locally Advanced | Yes | Unresectable at surgical exploration |
| 14 | Not Detected | Locally Advanced | Yes | Unresectable after NAC; transitioned to definitive chemoradiation |
| 16 | Not Detected | Borderline Resectable | Yes | Unresectable on diagnostic laparoscopy |
| 17 | Detected | Locally Advanced | Yes | Metastatic progression on NAC |
| 21 | Detected | Resectable | Yes | Metastatic progression |
| 30 | Detected | Borderline Resectable | Yes | Metastatic progression |
| 33 | Detected | Borderline Resectable | Yes | Metastatic progression |
| 36 | Detected | Resectable | Yes | Progression of disease |
| 44 | Not Detected | Borderline Resectable | Yes | Metastatic progression |
| 46 | Detected | Borderline Resectable | Yes | No carcinoma on any biopsies; transitioned to definitive chemoradiation |
| 48 | Not Detected | Borderline Resectable | Yes | Elected hospice care, surgery not pursued |
| 50 | Not Detected | Locally Advanced | No | Metastatic progression |
| 51 | Detected | Locally Advanced | Yes | Unresectable vascular involvement |
| 53 | Not Detected | Locally Advanced | Yes | Unresectable vascular involvement |
| 55 | Detected | Locally Advanced | Yes | Unresectable vascular involvement |
| 58 | Not Detected | Locally Advanced | Yes | Unresectable during surgery |
| 60 | Detected | Borderline Resectable | No | Metastatic progression |
| 63 | Detected | Borderline Resectable | Yes | Unresectable after chemoradiation |
| 64 | Detected | Borderline Resectable | Yes | Unresectable vascular involvement |
| 65 | Detected | Borderline Resectable | Yes | Unresectable at surgery |
| 72 | Detected | Locally Advanced | Yes | Metastatic progression |
| 76 | Detected | Borderline Resectable | Yes | Death during NAC |
| 81 | Detected | Borderline Resectable | Yes | Pathology showed adenosquamous carcinoma, surgery not pursued |
| 86 | Detected | Locally Advanced | Yes | Death during NAC |
| 89 | Not Detected | Borderline Resectable | Yes | Unresectable vascular involvement |
| 90 | Detected | Resectable | No | Metastatic progression |
| 93 | Detected | Borderline Resectable | Yes | Progression of disease |
| 95 | Detected | Borderline Resectable | Yes | Progression of disease |
| 98 | Not Detected | Locally Advanced | No | Unresectable vascular involvement |
| 100 | Not Performed | Locally Advanced | Yes | Death during NAC |
| 103 | Detected | Resectable | No | Death prior to NAC |
| 104 | Not Detected | Borderline Resectable | Yes | Metastatic progression |
| 105 | Detected | Borderline Resectable | Yes | Unresectable vascular involvement |
| 107 | Detected | Resectable | Yes | Metastatic progression |
| 108 | Not Detected | Resectable | Yes | Metastatic progression |
| 109 | Detected | Locally Advanced | Yes | Unresectable vascular involvement |
| 110 | Not Detected | Locally Advanced | Yes | Unresectable vascular involvement |
| 113 | Not Detected | Resectable | Yes | Elected hospice care, surgery not pursued |

*****Patient identifiers correspond to those shown in the oncoplot in Supplemental Figure 4.

Abbreviations: ID, identification; NAC, neoadjuvant chemotherapy

Supplemental Figure 1:  Flow diagram depicting the cohort formation process, outlining the exclusions and inclusion criteria for the study participants

Supplemental Figure 2: Sankey diagram showing patient enrollment, sample collection, treatment pathway, and ctDNA detection

Abbreviations: ctDNA, circulating tumor DNA; NAC, neoadjuvant chemotherapy

Supplemental Figure 3: Kaplan-Meier curve depicting OS by NGS assay: Tempus vs Predicine

Abbreviations: NGS, next-generation sequencing; OS, overall survival

Supplemental Figure 4: Oncoplot illustrating mutations identified by next-generation sequencing across all patients in the cohort. Filled boxes indicate the presence of specific mutations detected in each patient at any timepoint during the study.

Supplemental Figure 5a-f: Supplemental Figure 5a-b show Kaplan-Meier curves comparing OS based on the presence or absence of detectable total ctDNA by NGS after NAC and after resection. Supplemental Figures 5c–d present Kaplan–Meier curves comparing overall survival by *KRAS*-specific ctDNA detection at the same timepoints, while Supplemental Figure 5e–f display analogous curves stratified by *TP53*-specific ctDNA detection.

b

a

c

d

e

f

Abbreviations: ctDNA, circulating tumor DNA; KRAS, Kirsten rat sarcoma; NAC, neoadjuvant chemotherapy; NGS, next-generation sequencing; OS, overall survival

Supplemental Figure 6a-i: Supplemental Figure 6a-c show Kaplan-Meier curves comparing OS based on the presence or absence of detectable total ctDNA by NGS at diagnosis, after NAC, and after resection for patients receiving neoadjuvant FOLFIRINOX. Supplemental Figure 6d–f present Kaplan–Meier curves comparing overall survival by *KRAS*-specific ctDNA detection at the same timepoints, while Supplemental Figure 6g–i display analogous curves stratified by *TP53*-specific ctDNA detection.

c

b

a

d

f

e

i

h

g

Supplemental Figure 7a-h: Supplemental Figure 7a-c show Kaplan-Meier curves comparing OS based on the presence or absence of detectable total ctDNA by NGS at diagnosis, after NAC, and after resection for patients receiving neoadjuvant gemcitabine. Supplemental Figure 7d–e present Kaplan–Meier curves comparing overall survival by *KRAS*-specific ctDNA detection at diagnosis and post-resection; the post-NAC analysis could not be performed because no patients had detectable *KRAS* ctDNA at that timepoint. Supplemental Figure 7f–h display analogous curves stratified by *TP53*-specific ctDNA detection.

c

a

b

e

d

f

h

g

Supplemental Figure 8a-c: Figure 8a displays a boxplot of total ctDNA mVAF detected by NGS at three clinical timepoints: at diagnosis, after NAC, and after resection. Figures 8b and 8c present mVAF of KRAS-specific and *TP53*-specific ctDNA, respectively. Median values are annotated by each box.

c

b

a

**References**

1.	Finkle JD, Boulos H, Driessen TM, Lo C, Blidner RA, Hafez A, et al. Validation of a liquid biopsy assay with molecular and clinical profiling of circulating tumor DNA. NPJ Precis Oncol. 2021;5(1):63.

2.	Predicine. PredicineCARE™: CLIA validated cfDNA NGS assay for pan-cancer therapy selection and monitoring. 2025.
